# Effects of Pre-Encoding Stress on Brain Correlates Associated with the Long-Term Memory for Emotional Scenes

**DOI:** 10.1371/journal.pone.0068212

**Published:** 2013-09-05

**Authors:** Janine Wirkner, Mathias Weymar, Andreas Löw, Alfons O. Hamm

**Affiliations:** 1 Department of Biological and Clinical Psychology, University of Greifswald, Greifswald, Germany; 2 Center for the Study of Emotion and Attention, University of Florida, Gainesville, Florida, United States of America; The University of Queensland, Australia

## Abstract

Recent animal and human research indicates that stress around the time of encoding enhances long-term memory for emotionally arousing events but neural evidence remains unclear. In the present study we used the ERP old/new effect to investigate brain dynamics underlying the long-term effects of acute pre-encoding stress on memory for emotional and neutral scenes. Participants were exposed either to the Socially Evaluated Cold Pressure Test (SECPT) or a warm water control procedure before viewing 30 unpleasant, 30 neutral and 30 pleasant pictures. Two weeks after encoding, recognition memory was tested using 90 old and 90 new pictures. Emotional pictures were better recognized than neutral pictures in both groups and related to an enhanced centro-parietal ERP old/new difference (400–800 ms) during recognition, which suggests better recollection. Most interestingly, pre-encoding stress exposure specifically increased the ERP old/new-effect for emotional (unpleasant) pictures, but not for neutral pictures. These enhanced ERP/old new differences for emotional (unpleasant) scenes were particularly pronounced for those participants who reported high levels of stress during the SECPT. The results suggest that acute pre-encoding stress specifically strengthens brain signals of emotional memories, substantiating a facilitating role of stress on memory for emotional scenes.

## Introduction

Acute stress initiates various bodily adaptation processes to establish physiological homoeostasis and affects cognitive processes such as attention, learning and memory. The link between stress and cognitive functioning has been the focus of intensive basic and clinical research. Experiencing episodes of extreme stress can lead to mental disorders such as post-traumatic stress disorder (PTSD), which is characterized by severe impairment in cognitive functioning and memory (e.g., intrusive recollections and hyper-arousal) [1].

However, studies that investigated the effects of stress and elevated stress hormone levels on learning and memory in healthy populations have reported mixed findings [2]–[4]. Acute stress prior to memory retrieval can impair memory performance [5], [6]. Kuhlmann et al. found that free recall of previously learned negative and positive words, but not of neutral words, was impaired after experiencing a psychosocial stressor shortly prior to memory testing [6].

In contrast, acute stress and stress level glucocorticoid doses around the time of encoding have been shown to improve later memory performance for emotionally arousing events in humans and animals [4], [7], [8]. For instance, exposure to cold pressure stress immediately after encoding high arousing emotional and neutral IAPS pictures led to enhanced memory recall for emotional pictures, but not for neutral pictures one week later [9]. Similarly, Payne et al. found that pre-encoding psychosocial stress facilitated memory for an emotional story while recognition for the neutral episode was impaired. These discrepant findings for emotional and neutral materials have been discussed to result from differential effects of stress and stress hormones on brain regions involved in either attention or memory control [8]. Likewise, cortisol administration, shortly before picture viewing, enhanced later incidental memory performance for pleasant and unpleasant compared to neutral pictures [10]. Memory enhancement after pre-encoding stress was also shown for emotional words; conversely, memory for neutral words were impaired [11]. Additionally, studies with neutral stimulus materials found mixed results or even failed to show such memory enhancing effects [12], [13]; therefore suggesting that noradrenergic activation due to emotional arousal seems to be essential for the memory enhancing effects of pre-encoding stress [14]–[16].

A key advantage of ERPs compared to other brain imaging techniques is that they provide measures of neural activity with extraordinary time resolution in real time and thus makes them ideally suited to examine neural events responsible for human memory [17]. ERPs of the retrieval of previously encoded items have been traditionally studied in recognition memory tasks, where old and new items are presented. It has been repeatedly shown that ERPs elicited by recognized old items evoke more positive going waveforms than those elicited by correctly classified new items [18]–[20]. Early research suggests a dual-process model where recognition memory is assumed to be based on familiarity (i.e. the feeling of knowing an item), and recollection, characterized by detailed item recognition and supposed to require hippocampal involvement [21]. Linking the ERP data to the dual-process model of recognition memory the ERP old/new effect was separated into two topographically and temporally distinct components: an early effect over frontal electrode sites, peaking between 300 and 500 ms and a late centroparietal old/new effect starting at about 400 ms after stimulus onset. There is multiple evidence associating the late centroparietal old/new effect with recollective experience and hippocampus-dependent recognition [20], [22]–[24], whereas the assumption of the early frontal component reflecting familiarity processes is still under debate [17], [25].

Recent picture memory studies using longer retention intervals from 24 hours up to one year [26]–[30] suggest that the centroparietal old/new effect is modulated by emotional arousal, showing higher old/new differences for emotional pictures compared to neutral pictures, in accordance with better memory performance for these stimuli [31]. Moreover, the centroparietal effect for emotional contents is related to increased confidence [28], [29] and remember judgments, supporting the functional association of the parietal old/new difference with recollective experience and the role of recollection in emotional memory [32].

In the present study, we examined the influence of acute pre-encoding stress on brain dynamics associated with the long-term memory (two-week interval) for emotionally arousing and neutral scenes. In accordance with our previous ERP long-term recognition memory studies [26], [28], we predicted better memory performance for emotionally arousing compared to neutral pictures. For ERPs, we expected to find larger centro-parietal positivity for old, compared to new pictures during recognition, with larger ERP old/new differences for emotional relative to neutral pictures. If stress during encoding facilitates memory consolidation for emotionally arousing events [15] we further expected enhanced recognition and larger ERP old/new effects following acute pre-encoding stress selectively for emotionally arousing pictures. Recognition and brain potentials of neutral pictures, on the other hand, were expected to be unaffected or even impaired [11] by the stress manipulation.

## Materials and Methods

### Ethics Statement

Participants provided informed written consent for the protocol approved by the Review Board of the University of Greifswald and received financial compensation for participation. The study conforms with The Code of Ethics of the World Medical Association (Declaration of Helsinki) printed in the British Medical Journal (18 July 1964).

### Participants

Fifty-two healthy students (23 females) from the University of Greifswald (mean age: 23.0 years, range: 18–30, 4 left-handed, mean body-mass-index (BMI): 22.2, range 19–27 kg/m^2^) participated in the study. Exclusion criteria were checked in a standardized telephone interview and included smoking, current or lifetime diagnosis of mental disorders, medical conditions and medication intake within the prior three weeks, and during study participation. Participants were instructed to refrain from physical exercise, meals and caffeine intake within 3 h prior to the experimental sessions. Female participants reported a regular cycle with six subjects in follicular and 17 in luteal phase. All participants had normal or corrected-to-normal vision.

### Stress protocol and control condition

Participants were randomly assigned to either the stress or control condition. Groups did not differ for age (F_(1,51)_ = 1.1, p = .19), sex, handedness, BMI and menstrual cycle for females (all F_(1,51)_<1; see Table 1).

**Table 1:** Sample characteristics.

	Control	Stress
n	26	26
Mean (SEM) age [years]	23.6 (.61)	22.5 (.64)
Sex (male/female)	15/11	14/12
Female menstrual cycle		
follicular/luteal phase	2/9	4/8
Handedness		
left/right	24/2	24/2
Mean (SEM) BMI [kg/m^2^]	22.3 (.39)	22.0 (.34)

In the stress condition, participants (N = 26) were exposed to the Socially Evaluated Cold Pressure Test (SECPT) as described by Schwabe et al. [33]. Participants were monitored by a rather cold and unsociable experimenter and were asked to immerse their right hand, including the wrist, into ice water (temperature: 0–2°C) for 3 min (or until they could no longer tolerate it). During hand immersion, participants were videotaped, asked to look straight into the camera and told that video recordings would later be analyzed for facial expressions. Several studies have shown that the SECPT is an effective stress induction method that leads to significant elevations in autonomic arousal, salivary cortisol and subjective stress ratings [34]–[36]. Participants in the control condition (N = 26) immersed their right hand including the wrist for 3 min in warm water (35–37°C). They were neither videotaped nor monitored by an unfamiliar experimenter. To validate the efficacy of the SECPT, cardiovascular measures (heart rate and blood pressure) were recorded manually (using Riva-Rocci technique) immediately before (pre), during and after (post) SECPT or warm water test. Participants then rated on a scale from 0 (“not at all”) to 100 (“very much”) how stressful, painful and unpleasant the previous procedure was and how difficult it was to keep the hand immersed in the water.

### Stimulus materials

Stimuli consisted of 180 pictures (60 unpleasant, 60 neutral and 60 pleasant pictures) taken from the International Affective Picture Series (IAPS) [37] and the Emotional Picture Set (EmoPicS) [38] (IAPS and EmoPicS Numbers. Set 1: Unpleasant: 1019, 1220, 1300, 1932, 2352,2, 3019, 3064, 3102, 3110, 3150, 3180, 3190, 3191, 3195, 3530, 6210, 6212, 6313, 6560, 6571, 8480, 9042, 9230, 9301, 9490, 9561, 9599, 9902, 9910, 9921; Neutral: EP278, 2026, 2038, 2357, 2390, 2512, 2513, 2850, 2890, 5130, 5390, 5535, 5593, 5726, 5800, 7037, 7041, 7150, 7205, 7207, 7234, 7491, 7495, 7546, 7550, 7595, 7900, 7920, 9210, 9360; Pleasant: 1463, 1540, 1710, 1811, 2040, 2158, 2160, 2208, 2300, 4604, 4611, 4640, 4647, 4652, 4658, 4659, 4681, 5470, 5621, 5626, 8030, 8160, 8170, 8180, 8260, EP075, 8470, 8490; Set2: Unpleasant: 1052, 1201, 1304, 1726, 1931, 3015, 3051, 3062, 3100, 3101, 3140, 3160, 3225, 3250, 3261, 6260, 6370, 6410, 6563, 6821, 9008, 9440, 9520, 9560, 9570, 9600, 9622, 9630, 9635,1, 9908; Neutral: EP308, 2190, 2206, 2273, 2383, 2595, 2749, 2840, 2870, 2980, 5120, 5510, 5635, 5711, EP345, 5875, 6000, 7038, 7130, 7160, 7179, 7233, 7490, 7493, 7500, 7510, 7547, 7590, 7710, 9401; Pleasant: 1440, 1590, 1720, 1722, 2058, 2075, 2080, 2340, 2345, 4598, 4599, 4645, 4651, 4656, 4687, 4693, 4694, 4800, 5629, 8021, 8041, 8080, 8161, 8185, 8186, 8190, 8300, 8370, 8380). Two stimulus sets were carefully matched according to their normative hedonic valence and arousal ratings (see IAPS and EmoPicS norms for both sexes; set 1: mean hedonic valence = 2.6, 5.1 and 7.1; mean arousal = 6.1, 3.2 and 5.9 for unpleasant, neutral and pleasant pictures, set 2: mean hedonic valence = 2.6, 5.2 and 7.1; mean arousal = 6.0, 3.2 and 5.9). Additionally, both sets were matched for semantic categories (e.g., pictures of attack, mutilations, neutral people, objects, adventure, and erotic couples). The picture sets were counterbalanced during encoding so half of the sample viewed each of the two picture sets.

Each picture set consisted of 90 pictures (30 unpleasant, 30 neutral and 30 pleasant pictures, respectively). 21 pictures were added before (7 unpleasant, 7 neutral, 7 pleasant) and after (7 unpleasant, 7 neutral, 7 pleasant) picture presentation to avoid serial position effects on subsequent memory performance. These pictures were not included in the analyses.

In addition, individual hedonic valence and arousal ratings for all pictures in our sample were obtained to check for correspondence with normative ratings of the IAPS and EmoPicS. As expected, unpleasant pictures were rated as more unpleasant (Mean valence: 2.8) than neutral (Mean valence: 4.9; F_(1,51)_ = 475.08, p<.001) and pleasant (Mean valence: 6.8, F_(1,51)_ = 883.74, p<.001) pictures. Additionally, emotional pictures (pleasant, Mean arousal: 4.5; unpleasant, Mean arousal: 5.8) were rated as more arousing than neutral pictures (Mean arousal: 2.4; F_(1,51)_ = 349, p<.001). As in our previous study [36], there were no group differences between SECPT and control condition regarding hedonic valence (F_(1,25)_ = 2.6, p = .118) and arousal (F_(1,25)_<1) ratings. No differences were observed between both picture sets.

### Procedure

All experimental sessions took place in the afternoon between 1 and 5 pm. After participant's arrival at the laboratory, heart rate and blood pressure measurements were taken. Then, participants were exposed to either the SEPCT or control condition. Heart rate and blood pressure were measured during hand immersion. Participants then rated how stressful, painful and unpleasant the previous situation was and how difficult it was to keep the hand immersed in the water. In addition, heart rate and blood pressure were recorded again. During the following encoding session, 90 pictures were presented on a 20-inch computer screen for 3000 ms with a random inter-trial interval (ITI) of 2000, 2500 or 3000 ms. A 500 ms fixation cross preceded the onset of each picture to ensure that participants fixated the center of the screen. The pictures were presented in pseudorandom order for each participant with the restriction that no picture from the same valence category was presented in two consecutive trials. Participants were instructed to attentively watch the pictures and to avoid eye blinks and body movements during ERP measurement. No mention of a memory test was made (incidental encoding).

Two weeks after the encoding session participants returned to the lab for memory testing. After attaching the EEG electrodes, a recognition test was conducted during which 90 previously seen pictures (30 unpleasant, 30 neutral, and 30 pleasant pictures) were presented randomly intermixed with 90 new pictures that were matched for content, valence and arousal. Each picture was displayed for 3000 ms and preceded by a 500 ms fixation cross. Participants were instructed to attentively watch the pictures and to avoid eye blinks and body movements during ERP measurement. Following each picture, participants had to indicate whether they had seen the picture before or not, by pressing either a “yes” or “no” button. The assignment of left and right button presses to yes/no responses was counterbalanced across participants. After recognition, participants were asked to rate all previously seen pictures for their subjective hedonic valence and arousal using the SAM rating procedure [39].

### Electrophysiological recording

EEG signals were recorded continuously from 257 electrodes using an Electrical Geodesics (EGI) HydroCel high-density EEG system with NetStation software on a Macintosh computer. The EEG recording was digitized at a rate of 250 Hz, using the vertex sensor Cz as recording reference. Scalp impedance for each sensor was kept below 30 kΩ. All channels were bandpass filtered online from 0.1 to 100 Hz. Offline reduction was performed using EMEGS [40] and included lowpass filtering at 40 Hz, artifact detection, sensor interpolation, baseline correction, and conversion to the average reference [41]. Stimulus-synchronized epochs were extracted from 100 ms before to 1200 ms after picture onset and baseline corrected (100 ms prior to stimulus onset).

ERPs were computed for the three emotional picture categories (unpleasant, neutral and pleasant) in each experimental group (stress vs. control). Only trials with correct responses (correctly recognized old pictures and correctly classified new pictures, respectively) were included in ERP averages.

### Data analysis

**Figure 1:**
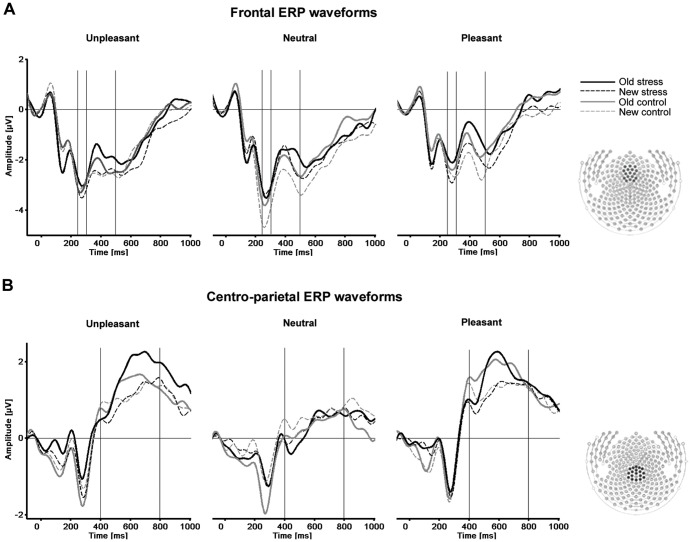
Grand average ERPs waveforms at frontal (A) and centro-parietal (B) sensor clusters for old (thick line) and new (dotted line) unpleasant, neutral and pleasant pictures in stressed (black lines) and control (grey lines) participants.

To identify sensor clusters representative for the old/new effect, visual inspection and single-sensor waveform analyses were used in concert. On the basis of this inspection, ANOVAs, including the factors Emotion (unpleasant vs. neutral vs. pleasant), Memory (old vs. new) and Group (stress vs. control), were calculated for each time point and each individual sensor [28]. Based on these results and guided by previous studies [22], [26], [28], two time windows and electrode clusters (Figure 1) were selected for further statistical analyses. An electrode cluster over frontal sites (including EGI sensors 5, 6, 7, 8, 13, 14, 15, 16, 17, 21, 22, 23, 24, 28, 29, 198 and 207) was selected for the early time window (300–500 ms) and a cento-parietal electrode cluster (including EGI sensors 45, 53, 79, 80, 81, 88, 89, 90, 100, 101, 129, 130, 131, 132, 142, 143, 144 and 257) was selected for the late time window between (400–800 ms).

Mean ERP amplitudes of both scalp clusters in the corresponding time windows were analyzed separately using an ANOVA involving the within-subject factors Emotion (unpleasant vs. neutral vs. pleasant) and Memory (old vs. new) as well as the between-group factor Group (stress vs. control).

In addition, group differences were observed for old and new neutral and unpleasant pictures in the time window between 250 and 300 ms over the frontal electrodes (see Figure 1). Mean N200 amplitudes were analyzed using an ANOVA involving the within-subject factors Emotion (unpleasant vs. neutral vs. pleasant) and Memory (old vs. new) and the between-group factor Group (stress vs. control).

For behavioral performance, hit rates, false alarm rates and the discrimination index Pr for recognition were analyzed using an ANOVA involving the factors Emotion (unpleasant vs. neutral vs. pleasant) and Group (stress vs. control). All analyses were conducted with SPSS 19.0 (IBM, Armonk, NY, USA).

For effects involving repeated measures, the Greenhouse-Geisser procedure was used to correct violations of sphericity.

## Results

### Stress ratings and cardiovascular responses to the SECPT/warm water control

Autonomic and subjective measurements indicated a successful stress induction by the SECPT (see Table 2).

**Table 2:** Subjective stress ratings and autonomic measures during and after the SECPT/warm water control condition.

	Control	Stress
*Stress ratings*		
Stressful	6.9 (2.3)	45.8 (5.2)**
Painful	3.8 (1.8)	59.2 (5.1)**
Unpleasant	8.5 (3.4)	61.5 (5.4)**
Hard to tolerate	9.6 (3.7)	57.7 (5.0)**
*Heart rate (bpm)*		
During hand immersion	63.7 (1.5)	71.6 (2.5)*
After hand immersion	63.7 (1.8)	64.9 (1.8)
*Systolic blood pressure (mmHg)*		
During hand immersion	121.5 (1.3)	129.7 (1.6)**
After hand immersion	121.1 (0.9)	118.6 (1.5)
*Diastolic blood pressure (mmHg)*		
During hand immersion	79.6 (0.6)	89.5 (1.2)**
After hand immersion	79.3 (0.7)	77.8 (1.0)

Subjective assessments were measured using a scale from 0 (“not at all”) to 100 (“very much”). Data represent means (SEM). Bold indicates significantly higher values in stress compared to control group (*p<.05, **p<.001).

#### Stress ratings

As expected, participants rated the hand immersion in the SECPT condition as significantly more stressful (F_(1,50)_ = 46.29, p<.001), painful (F_(1,50)_ = 104.94, p<.001) and unpleasant (F_(1,50)_ = 67.63, p<.001) than the participants in the warm water control condition. In addition, hand immersion was harder to tolerate in the stress group (F_(1,50)_ = 59.25, p<.001) compared to the warm water control group.

#### Heart rate and blood pressure

Exposure to the SECPT also resulted in significantly stronger increases in heart rate (Time×Group, F_(2,50)_ = 4.14, p<.05), systolic (Time×Group, F_(2,50)_ = 69.85, p<.001) and diastolic (Time×Group, F_(2,50)_ = 8.69, p<.01) blood pressure compared to the control group. Moreover, during hand immersion, participants in the SECPT group showed significantly elevated autonomic reactions (heart rate: F_(1,50)_ = 6.80, p<.05; systolic blood pressure: F_(1,50)_ = 15.48, p<.001; diastolic blood pressure: F_(1,50)_ = 50.63, p<.001) compared to the control group (see Table 2). No significant group differences were observed immediately before and after hand immersion, supporting the view that the observed group differences were specifically induced by the stress test.

### Recognition: Behavioral data

Table 3 lists memory performance for old and new pictures as a function of picture content and experimental group.

**Table 3:** Memory: Behavioral Data.

	Control	Stress
Hit rate		
Unpleasant	.84 (.02)	.78 (.03)
Neutral	.66 (.04)	.58 (.04)
Pleasant	.78 (.03)	.70 (.03)
FA rate		
Unpleasant	.15 (.02)	.17 (.02)
Neutral	.16 (.02)	.20 (.02)
Pleasant	.19 (.02)	.20 (.02)
Pr		
Unpleasant	.69 (.02)	.61 (.03)
Neutral	.51 (.03)*	.39 (.03)
Pleasant	.58 (.02)	.50 (.03)

Numbers represent means for hit and false alarm rates and discrimination Pr for each picture type (SEM). Bold indicates significantly higher values in control compared to stress group (*p<.05).

#### Recognition memory performance

As expected for hit rate (hr), a main effect of Emotion (F_(2,100)_ = 42.44, p<.001) indicated that pictures with emotional contents were better remembered than neutral pictures. Unpleasant pictures (Mean (hr) = .81) were better recognized than pleasant (Mean (hr) = .74) pictures (F_(1,50)_ = 24.36, p<.001). Consistent with hit rate, correct discrimination (Pr) between old and new pictures was better for emotional compared to neutral pictures (F_(2,100)_ = 47.66, p<.001). False alarm rates differed between emotional contents (F_(2,100)_ = 4.21, p<.05) and were slightly higher for pleasant compared to unpleasant pictures. No group effects were observed for false alarms (F_(1,50)_<1, p = .434) or hit rates (F_(1,50)_ = 3.21, p = .079). In contrast, a main effect for group (F_(1,50)_ = 6.94, p<.05) indicated that picture discrimination (Pr) was overall better in the warm water control compared to the stress group. Post hoc tests showed that discrimination was better for neutral pictures in the controls compared to stress group (F_(1,50)_ = 7.01, p<.05); however these group differences did not occur for emotionally arousing pictures (F1,50) = 2.83, p = .10).

### Recognition: ERP data

Figure 1 illustrates the grand average ERPs for correctly recognized old and new pictures of two representative sensor clusters as a function of picture content (unpleasant, neutral and pleasant pictures) and group (stress vs. control).

#### N200

In the time window from 250 to 300 ms, new pictures evoked a larger ERP negativity than old pictures (Memory: F_(1,50)_ = 9.18, p<.01) over frontal sensor sites. A significant interaction (Emotion×Memory×Group: F_(2,100)_ = 3.228, p<.05) indicated that novel neutral pictures prompted a larger N200 than correctly recognized old neutral pictures in the control group (F_(1,25)_ = 4.50, p<.05). This effect did not occur in the SECPT group (F_(1,25)_<1, p = .731). In contrast, new unpleasant pictures prompted a larger negativity than correctly recognized old unpleasant pictures in the SEPCT group but not in the controls (F_(1,25)_ = 4.32, p<.05; Control group: F_(1,25)_<1, p = .718). For pleasant scenes, the N200 in response to novel pictures, compared to old, did not differ between stress and control group (Memory×Group: F_(1,50)_ = 1.20, p = .278).

#### Early old/new effect

In the early time window from 300 to 500 ms, correctly recognized old pictures prompted more positivity, relative to correctly classified new pictures over frontal (Memory: F_(1,50)_ = 39.30, p<.001), but not over centro-parietal sensor sites (Memory: F_(1,50)_<1, p = .770). No main effects of group or any interactions were significant over frontal (F_(1,50)_<1, p = .542) and centro-parietal sensors (F(1,50)<1, p = .450) in the early time window.

#### Late old/new effect

In the late time window between 400 and 800 ms, there was a prominent old/new effect (Memory: F_(1,50)_ = 19.45, p<.001) over frontal electrodes but no interaction with emotion (F_(2,100)_<1, p = .50). No group differences were observed between SECPT and control group (F_(1,50)_<1, p = .943).

**Figure 2:**
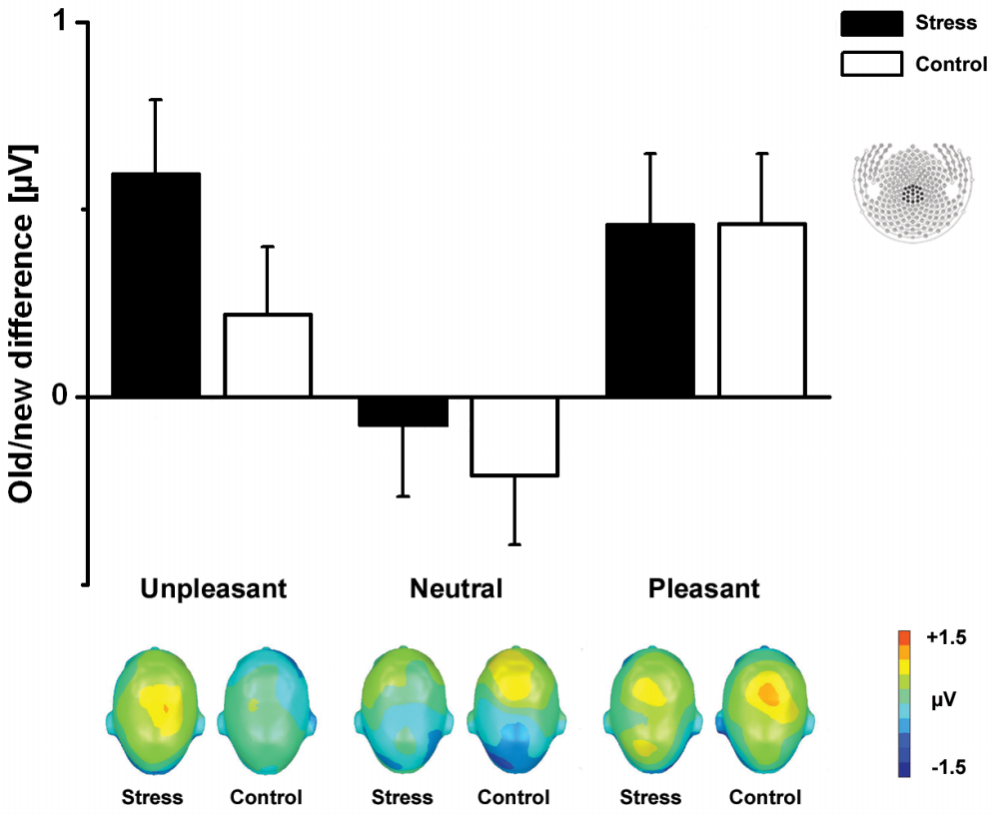
ERP old/new differences. The upper section shows the ERP old/new effect (old minus new) of the mean amplitudes recorded over the centro-parietal cluster in the 400–800 ms time window for stress and control group. Error bars represent standard error of the means (SEM). The lower section displays the corresponding scalp topographies of the ERP difference separately for the three picture categories (top view) and group.

For centro-parietal sensors, correctly recognized old pictures showed greater ERP positivity than new pictures (Memory: F(1,50) = 13.45, p<.01). Notably, this old/new difference was modulated by emotion (Emotion×Memory: F_(2,100)_ = 5.46, p<.01) with emotional pictures showing larger old/new differences than neutral pictures (F_(1,50)_ = 9.06, p<.01) (see Figure 2). The old/new difference for unpleasant pictures was significantly larger in the stress group compared to the non-stressed control group (F_(1,25)_ = 4.45, p<.05). These group differences were not observed during correct recognition of neutral or pleasant pictures. The mean amplitude changes during correct recognition of old and new pictures as a function of emotion and group are listed in Table 4.

**Table 4:** ERPs for correctly classified old and new pictures averaged over centro-parietal sensors (400–800 ms).

	Control		Stress		High stress		Low stress	
	Old	New	Old	New	Old	New	Old	New
Unpleasant	1.39	1.17	1.67	1.07	1.87	.93	1.46	1.21
	(.19)	(.23)	(.22)	(.18)	(.35)	(.28)	(.27)	(.23)
Neutral	.38	.59	.32	.39	.20	.34	.43	.44
	(.19)	(.20)	(.21)	(.15)	(.36)	(.19)	(.25)	(.25)
Pleasant	1.77	1.31	1.65	1.19	1.86	1.05	1.44	1.33
	(.20)	(.20)	(.20)	(.22)	(.27)	(.34)	(.29)	(.30)

Data represent means in µV (SEM).

#### Experienced stress and the centro-parietal ERP old/new effect

**Figure 3:**
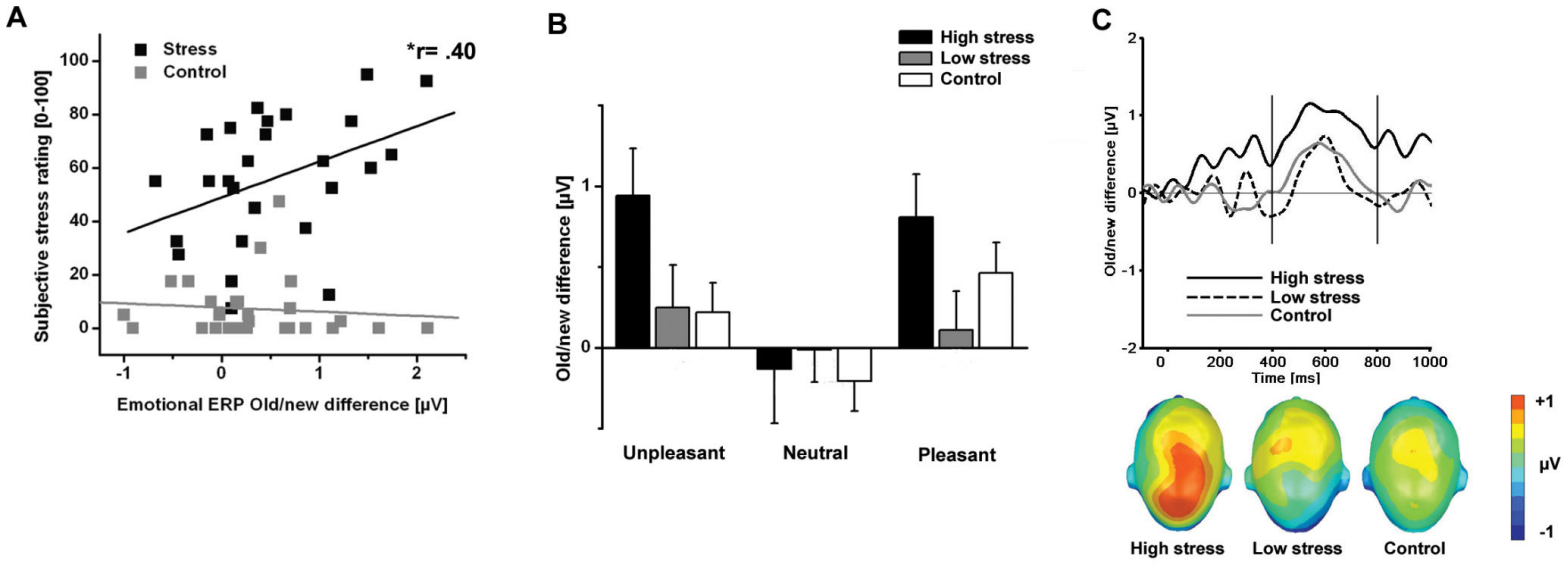
Subjective stress and centro-parietal ERP old/new effect. A. Experienced stress predicts enhanced centro-parietal ERP old/new difference for emotional pictures (400–800 ms) in the stress group. Correlations between (averaged) subjective stress ratings and centro-parietal ERP old/new effect (400–800 ms) for emotional pictures in both experimental groups (stress vs. controls). B. ERP old/new differences averaged over centroparietal sensors (400–800 ms) for unpleasant, neutral and pleasant pictures in high and low stressed participants and control group. Error bars indicate SEM. C. ERP difference waveforms (old-new) averaged over centroparietal sensors for emotional pictures in high stressed (black line), low stressed (dotted line) and control (grey line) participants. The lower section displays the corresponding scalp topographies of the ERP difference separately for the three groups.

To further examine the relationship between acute pre-encoding stress and memory, correlational and median split analyses were conducted. As a measure of overall experienced stress, the mean average of the four subjective stress ratings (stressful, painful, unpleasant and tolerance difficulty) was calculated. Significant correlations were observed between the reported stress level and the centro-parietal old/new effect (difference score: old minus new) for emotionally arousing pictures (Pearson correlation: Early time window: r = .51, one-tailed, p<.001; Late time window: r = .40, one-tailed, p<.05, see Figure 3A), but not for neutral ones in the SECPT group (Pearson correlation for unpleasant pictures: Early time window: r = .38, one-tailed, p<.05; Late time window: r = .32, one-tailed, p = .056 and Pearson correlation for pleasant pictures: Early time window: r = .36, one-tailed, p<.05; Late time window: r = .266, one-tailed, p = .094). As expected, no significant correlations between these variables were observed in the control group. Also no significant correlations were observed between reported stress levels and the frontal ERP old/new difference in both time windows.

A median split was performed for the overall reported stress level in the SECPT group (Median = 57.50), in which participants were divided into a high stress (Mean: 75.0) and low stress group (Mean: 37.11). Figure 3 (B and C) illustrates the influence of experienced stress (high vs. low) on the late centro-parietal old/new differences for emotionally arousing and neutral pictures.

## Discussion

In the present study, we examined the influence of acute pre-encoding stress on brain potentials of long-term memory for emotional and neutral scenes. Correct recognition of previously seen pictures evoked enhanced positivity over centro-parietal sensor sites in the late time window (400–800 ms) compared to viewing of new pictures. This late old/new difference was more pronounced for emotional picture contents. Interestingly, although memory performance was not generally facilitated by stress exposure, we found that acute pre-encoding stress specifically enhanced the late centro-parietal old/new effect for emotional (unpleasant) pictures. Furthermore, participants experiencing high levels of stress showed higher ERP old/new differences for emotionally arousing (unpleasant and pleasant) pictures, but not for neutral pictures. Taken together, pre-encoding stress prompts an enhanced old/new effect during recognition of emotional pictures that varies with individual stress levels.

Correct recognition of previously presented compared to correctly rejected new pictures was associated with enhanced ERP positivity over centro-parietal sensor sites (400–800 ms). This late old/new difference over posterior sensor sites has been reliably described as an electrophysiological correlate reflecting retrieval processes with specific episodic recollection [20] because it is modulated by depth of processing, correct source memory, “remember” and high confidence judgments [27], [28], [42]. As in earlier studies using longer retention intervals, memory performance was better for emotional compared to neutral contents [28], [29], [31], [43]. Moreover, enhanced memory for emotional pictures was reflected in greater ERP old/new differences during recognition of emotional compared to neutral pictures over centro-parietal sensor sites in the late time window, suggesting that emotional stimuli are better recollected than neutral stimuli [19], [27], [28], [30], [44].

Previous work has indicated that stress influences learning and memory processes depending on the exact timing of the stressor [45]. When experienced in the context and around the timing of the learning episode, acute stress has been suggested to promote selective attention processes and to further enhance memory consolidation, particularly for emotional contents [7], [9], [10], [15], [36], [46]. Overall, memory performance was not better in the stress, compared to control, group. In contrast, picture discrimination was significantly better for participants receiving the warm water relative to participants receiving the social and cold pressor stress, an effect that was most reliable for neutral contents. These data replicate previous findings which demonstrate that administration of stress and stress hormone doses around the time of encoding impair memory performance for neutral stimuli [11], [12]. Similar results have been described by Payne et al., where recognition memory performance for a neutral slide show was selectively impaired in stressed subjects [8]. Payne et al. suggested that pre-encoding stress exposure preserves or even enhances emotional aspects of an episode whereas memory for neutral information is disrupted; therefore pointing to a qualitatively different memory formation under stress experience [7]. Recent data suggest that reduced memory for neutral events after acute stress might be related to changes in adrenergic and noradrenergic signaling in hippocampus and midbrain circuits during memory formation [47], [48].

Even though the ERP old/new effect for neutral pictures did not differ between stressed and control participants, we found that new neutral pictures prompted an enhanced negativity (250 to 300 ms) compared to old neutral pictures in control participants. A fronto-central N200 has been discussed as an ERP component reflecting perceptual novelty during information processing, because it is elicited when a perceptual mismatch between the repetitive standard and an infrequent target is detected or when visual stimuli with novel features are presented [49]–[51]. No differences between neutral old and novel pictures were observed in stressed participants indicating that acute stress during encoding might affect later visual novelty detection (because of less specific memory representation) for neutral contents [47].

Although memory performance for emotional picture contents was not enhanced in the stress group on the behavioral level, we observed enhanced late centro-parietal ERP old/new differences for unpleasant pictures following pre-encoding stress exposure, indicating that the neural signature of the memory trace is enhanced for unpleasant stimuli encoded in the context of stressful experiences [15], [46]. Collapsing recollection and familiarity based answers during our recognition task could have prevented better behavioral memory performance for emotionally arousing pictures after pre-encoding stress. We suggest that stress might enhance recollection based recognition for emotionally arousing pictures as reflected in the ERP data. Different behavioral measures to tap recollection and familiarity (remember/know judgments, confidence ratings) could be helpful for disentangling the differential effects of acute stress on later behavioral memory performance.

Another line of research has described enhanced memory based on mood-congruency [52], in which affective stimuli that are encoded in congruent with a current mood state are better remembered than incongruent stimuli [53]. Consistent with this model, experiencing an unpleasant painful cold pressor stress might selectively enhance memory for unpleasant cues only, as indicated by the larger ERP old/new difference. On the other hand, additional correlation and median split analyses revealed that the enhanced ERP old/new difference was not selectively related to unpleasant scenes, since it was also found for pleasant materials. Participants reporting more stress during SECPT exposure showed enhanced ERP old/new difference amplitudes for emotionally arousing contents, but not for neutral ones. This finding suggests that, if the stress experience is intense enough, pre-encoding stress specifically facilitates episodic retrieval for emotionally arousing materials. Recent ERP and fMRI studies suggest that stress increases perceptual vigilance [36], [54]–[59] that might foster memory consolidation, particularly for emotional stimuli [60]. Replicating previous data [36], men showed larger late positive potentials (LPPs) during viewing of unpleasant stimuli following the cold pressor stress; therefore supporting the interpretation of more elaborative processing after stress. Women did not show this enhancement in the LPP. An elaborate discussion of these sex differences would, however, go beyond the scope of the current manuscript.

The amygdala is assumed to be the key brain structure for mediating stress effects on attentional networks and memory formation, interacting with several brain structures [such as the primary visual cortex, the prefrontal cortex, and the hippocampus] triggered by locus coeruleus-originating noradrenaline innervation [60], [61]. Corroborating these assumptions, van Marle et al. found enhanced connectivity between amygdala and locus coeruleus activation during resting state after administering a short psychological stressor, suggesting a prolonged state of hypervigilance after stress that may facilitate salience and memory formation [56]. The present study did not test whether the findings for the ERP old/new effect during retrieval was mediated by stress- induced changes in noradrenaline or glucocorticoid activity during memory formation; however, the SECPT has been used in previous studies as an efficient stress induction method that leads to significant elevations in both autonomic arousal and salivary cortisol [33], [35]. Furthermore, the ERP old/new effect of unpleasant pictures has been related to sympathetic activation during encoding [26], making it feasible that the combined action of noradrenaline and glucocorticoids on brain systems of attention and memory formation led to later changes in the ERP old/new effect in the present study.

From an evolutionary perspective, remembering the emotionally arousing aspects of a stressful experience is important for survival. But, experiencing highly arousing, life-threatening episodes under conditions of extreme stress can result in exceptionally strong, over-consolidated traumatic (fear) memories (re-experienced in flashbacks or nightmares). These memories often lack the integration of specific neutral context information, and thus may lead to the development of Posttraumatic Stress Disorder (PTSD) and an overgeneralization of fear [1], [48]. Interestingly, the individually perceived intensity of the traumatic event has been deliberated in the current DSM-5 debate to play an important role in the development of PTSD [62], [63].

To summarize, acute exposure to stress significantly increased the late centro-parietal old/new effect during retrieval of emotionally arousing pictures. Moreover, this effect was most pronounced in participants reporting high subjective stress experience. These findings suggest that recollection of emotional memories seems to be particularly facilitated when the stressful event around the time of encoding is also evaluated as intense, stressful and unpleasant.
